# Multicomponent Exercise Program to Improve the Immediate Sequelae of COVID-19: A Prospective Study with a Brief Report of 2-Year Follow-Up

**DOI:** 10.3390/ijerph191912396

**Published:** 2022-09-29

**Authors:** Lidia Martínez Rolando, Jorge Hugo Villafañe, Soledad Cercadillo García, Ana Sanz Argüello, Marta Villanueva Rosa, Eleuterio A. Sánchez Romero

**Affiliations:** 1 Department of Physiotherapy, Faculty of Sport Sciences, Universidad Europea de Madrid, 28670 Villaviciosa de Odón, Spain; 2 Musculoskeletal Pain and Motor Control Research Group, Faculty of Sport Sciences, Universidad Europea de Madrid, 28670 Villaviciosa de Odón, Spain; 3 Department of Physiotherapy, Faculty of Health Sciences, Universidad Europea de Canarias, 38300 Santa Cruz de Tenerife, Spain; 4 Musculoskeletal Pain and Motor Control Research Group, Faculty of Health Sciences, Universidad Europea de Canarias, 38300 Santa Cruz de Tenerife, Spain; 5 Rey Juan Carlos University Hospital of Móstoles, 28933 Madrid, Spain; 6 IRCCS Fondazione Don Carlo Gnocchi, 20141 Milan, Italy

**Keywords:** COVID-19, SARS-CoV-2, rehabilitation, therapeutic exercise, pulmonary rehabilitation

## Abstract

COVID-19 placed teams of professionals in a hostile and unfamiliar environment where the lack of knowledge of its pathology led to the adaptation of programs used so far for other conditions to try to address the immediate sequelae of COVID-19 infection. That is why the aim of this study was to assess the effects of a multicomponent exercise program (MEP) in improving cardio-respiratory performance, health status, disability due to dyspnea, aerobic capacity and endurance, and the immediate sequelae of COVID-19. Thirty-nine patients referred from different hospital services were included in this study. An intervention of seven weeks with sessions twice a week was carried out, where patients underwent intervallic training sessions followed by strengthening exercises and individualized respiratory physiotherapy exercises. The results of this study show a significant improvement in cardio-respiratory performance, health status, disability due to dyspnea, and aerobic capacity and endurance after intervention; and an increase in health status and reduction in disability due to dyspnea at the 2-year follow-up. In addition, none of the patients had any adverse effects either pre-post treatment or at the 2-year follow-up. Individualized and monitored MEP in survivors of COVID-19 showed positive effects in a pre-post evaluation and the 2-year follow up, improving the immediate sequelae of post-COVID-19 patients. This highlights the importance of the professional background of the rehabilitation teams in adapting to an unknown clinical environment.

## 1. Introduction

COVID-19 was declared a pandemic on 11 March 2020 by WHO (World Health Organization) due to its levels of spread and the severity of the situation; the effects of this condition have been visible from the very beginning and are still present [1,2]. Since the beginning of the pandemic, about 300 million cases have been reported and more than 5 million deaths have been confirmed [3]. Approximately 80% of those who have suffered from the disease have had mild symptoms; however, a significant proportion have suffered important multisystemic symptoms that resulted in an increase in admissions to the ICU (Intensive Care Unit) requiring a multidisciplinary approach from the acute phase [1,3,4,5,6,7,8].

As for the diagnosis, in addition to the clinical features detailed below that help to determine a possible infection, there are three diagnostic tools that can aid in the detection and confirmation of active infection. Therefore, the adequacy not only of the treatment but also of the sanitary clothing for the care of these patients is important. These diagnostic tools are the PCR (this is a molecular test), the antigen test (this detects viral proteins) and the antibody test (this detects the host’s response to infection or vaccination) [9]. Among the most frequent symptoms associated with this infection are fever, headache, musculoskeletal pain associated or not with fatigue, cough, pneumonia and dyspnea; other less frequent symptoms affecting other systems such as the digestive system (nausea, diarrhea…) may also be found [3]. The degree of involvement of these is dependent, among other factors, on the previous health status of the patient and therefore on the comorbidity associated with the individual [10]. Although the medium and long-term sequelae of this infectious process are unknown, the precedent of other epidemics caused by other coronaviruses shows that musculoskeletal and respiratory sequelae, among others, will occur in patients affected by COVID-19 [1,6,7].

The rapidity of infections led Spanish governmental institutions to declare a state of alarm that led to the imposition of home isolation and therefore limited the ability to leave the home, which was restricted to activities considered to be essential; therefore, patients who require hospital admission could not be accompanied by their closest and dearest, making health personnel their only support at the bedside, who were tasked with establishing a link beyond the professional [2]. 

The professionals were faced with a hitherto unknown clinical environment where the lack of knowledge of the pathology led the rehabilitation teams to establish individualized multicomponent programs according to the clinical condition of patients based on their training and previous clinical experience [5,11,12]. The extensive background of cardiorespiratory physiotherapy teams in the treatment of pathologies requiring functional retraining such as chronic obstructive pulmonary disease (COPD) or myocardial infarction led to the adaptation of these widely used multicomponent exercise programs that are described as the most effective interventions for improving effort tolerance in patients with respiratory diseases [13,14,15]. 

Similarly, current evidence shows that multicomponent exercise programs (MEP) are effective in reducing post-COVID-19 sequelae with faster recovery, which facilitates an improvement in the autonomy, functionality and quality of life of patients [1,16,17,18]. Furthermore, considering that global healthcare spending is expected to continue to grow, and that the available budget in countries is uneven, the development of economical and effective therapies such as the MEP is well suited to address many of the post-COVID-19 neuromusculoskeletal sequelae in the case of patients living in more vulnerable countries [19]. 

Therefore, the aim of this study was to assess the effects of an MEP on improving cardio-respiratory performance, health status, disability due to dyspnea, aerobic capacity and endurance, and the immediate sequelae of COVID-19.

## 2. Materials and Methods

### 2.1. Study Design

A prospective study was conducted in the rehabilitation ward of Rey Juan Carlos Hospital of Móstoles, Madrid, between June 2020 and December 2021, with telephone follow-up in May 2022. The procedure was conducted according to the Declaration of Helsinki and all subjects signed an informed consent form prior to participation in the study. The study was approved by the Research Ethics Committee of the Fundación Jiménez Díaz, Madrid (10 November 2020). 

### 2.2. Study Population

Thirty-nine patients referred from the ICU, Pneumology, Internal Medicine and Rehabilitation Services of Rey Juan Carlos Hospital were included, taking into account the following inclusion criteria: patients over 18 years of age, with a previous diagnosis of COVID-19 infection who required hospital admission, who at the time of starting the training presented a negative PCR test (polymerase chain reaction test) and decided to voluntarily enter the program; patients who presented some degree of functional impairment at the point of hospital discharge such as dysfunction/atrophy of the peripheral muscles and/or respiratory muscles. For exclusion criteria, the following were not included: patients with symptoms suggestive of active COVID-19 infection, comorbidities in the acute phase, decompensated cardiovascular pathology such as arterial hypertension (diastolic blood pressure above 100 mmHg and systolic pressure above 170 mmHg), acute respiratory pathology such as decompensated COPD (Chronic Obstructive Pulmonary Disease) with oxygen saturation below 90%, pulmonary thromboembolism; osteoarticular involvement preventing cyclergometer training; moderate/severe cognitive impairment and/or other symptoms such as uncontrolled diffuse pain, general fatigue, chest pain, severe cough and fever [20,21]. 

### 2.3. Outcomes Measures

All patients had a consultation with the rehabilitation physician who performed an anamnesis collecting data on the baseline situation prior to admission (work situation, sporting and social activity, personal history, and habits) and a comprehensive physical, respiratory and osteoarticular examination. Before starting the program, the physiotherapist team measured the following variables: 

Dyspnea using the modified Medical Research Council dyspnea scale (mMRC). This scale assesses dyspnea in activities of daily living scored from 0 (absence of dyspnea during intense exercise) to 4 (dyspnea prevents the patient from leaving the house or appears with tasks such as dressing or undressing) [20,22]. 

Quality of life was assessed according to the Short-Form 36 Questionnaire (SF-36) and COPD Assessment Test (CAT). SF-36 is an instrument used to assess health-related quality of life that evaluates eight spheres (physical functioning, role physical, bodily pain, general health, vitality, social functioning, role emotional and mental health) scored from 0 to 100, where 100 is equivalent to no disability and 0 is equivalent to maximum disability. The calculation of the score leads to the extraction of five health states: (1) Much better now than a year ago; (2) slightly better now than a year ago; (3) about the same as a year ago; (4) slightly worse now than a year ago; and (5) much worse now than a year ago [23]. CAT is an eight item questionnaire used to assess cough, sputum, chest tightness, breathlessness, activity limitation, confidence leaving home, sleep and energy which are scored from 0 to 4 in each item, 0 corresponds to the least affected and 5 to the most affected [24]

Exercise capacity was measured with a six-minute walking test (6MWT), for which the results of distance covered, oxygen saturation, heart rate (at the beginning, at the end and two minutes after the end of the test), blood pressure (at the beginning and two minutes after the end of the test), and Modified Borg Dyspnea Scale (at the end of the test) were collected [15,25,26]. This is a submaximal exercise test which consists of a patient walking for six minutes along a 30 m corridor with two cones marking the distance to be covered while being given a series of cues [17,21,25,26,27,28].

Aerobic capacity was measured with the Steep Ramp Test (SRT) of the Ergoline Program and the number of stops during the test were noted. Measurements were performed with a calibrated cyclergometer up to volitional maximal exertion; the attained peak work rate is the main outcome used to determine the training intensity [29]. 

### 2.4. Multicomponent Physical Exercise Intervention

**Figure 1:**
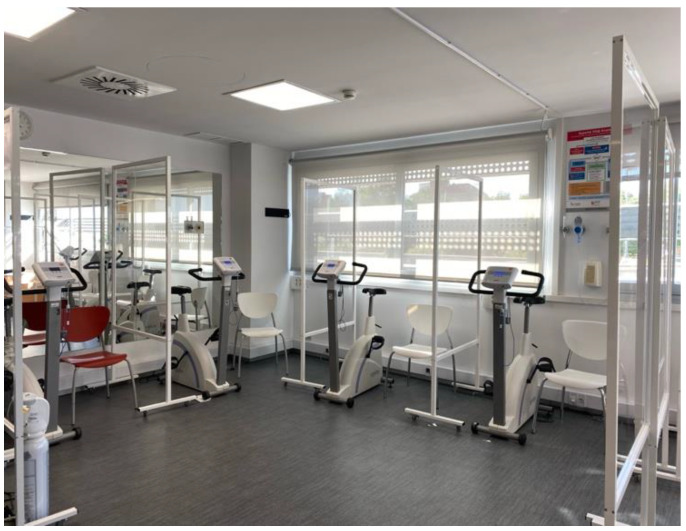
Rehabilitation ward of Rey Juan Carlos Hospital in compliance with COVID normative.

The intervention lasted seven weeks with sessions conducted twice a week for a total of 14 sessions. There were two groups per week with three patients in each group to comply with COVID normative protocols (interpersonal distance of one and a half meters delimited by methacrylate partitions and approved surgical mask) (Figure 1). 

Patients, prior to the start of training, had taken their usual medication and had a supply of water to ensure hydration; those patients using oxygen therapy were asked to perform the training with their usual guidelines. In each session, an initial and final measurement of constants was taken (temperature, blood pressure, saturation, electrocardiographic recording and glycemia in diabetics). Additional blood pressure and dyspnea perception results using the Borg Scale were obtained 20 min after the beginning of the training and saturation and heart rate were monitored throughout training. 

To establish the parameters of the cycloergometer interval training program, the SRT was performed beforehand, in which the patient pedaled for 3 min at 0 watts load at 50–60 cycles per minute. After the first 3 min, the load was increased by 25 watts every 10 s and the patient was asked to maintain the indicated cadence. The test ended when the patient could not maintain the indicated cadence, the saturation dropped below 90% or the heart rate reached its maximum theoretical heart rate.

This cycloergometer interval training had a duration of forty minutes where the first five and the last five minutes were performed at a constant load of 5% of its maximum load corresponding to the ’warm-up’ and the ´return to calm´ phase. The 30 min of intervallic work was distributed as follows: twenty seconds until reaching 60% of their maximum load in the first seven sessions (adding five watts each day) and until reaching 80% of their maximum load in the last session (14th session). Seventy seconds of rest at 20% of their maximum load was reserved.

**Figure 2:**
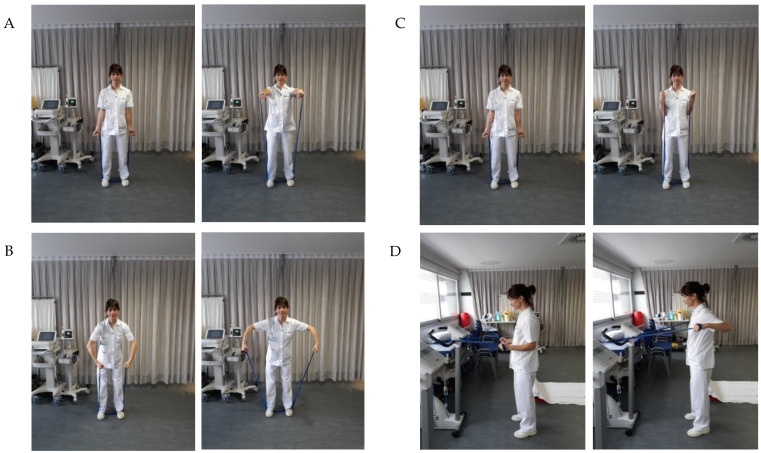
(**A**–**D**). Exercise protocol of upper limbs performed by patients. These exercises were designed to improve the strength of the upper limb. Anterior deltoids (**A**), middle deltoids (**B**), biceps (**C**) and dorsal muscles (**D**).

At the end of the sessions, strengthening exercises of the lower limbs (quadriceps, hamstrings, and gluteus muscles) and upper limbs (biceps, anterior and middle deltoids, and dorsal muscles) were performed with elastic bands; we adapted the resistance according to the characteristics of the patients. (Figure 2A–D). Additionally, individualized respiratory physiotherapy exercises (diaphragmatic stimulation, positive expiratory pressure exercises, alveolar recruitment and strengthening of inspiratory muscles) were taught. The necessary material and written guidelines with visual support were provided to encourage patients’ autonomy at home by creating an exercise habit. 

Two weeks after the study was completed, a new measurement of all the variables described above was performed and twenty-three months after the start of the intervention, an informal telephone interview was conducted to determine the status of patients by asking them about adherence to treatment, persistent signs and symptoms, return to work and limitations in daily life. 

### 2.5. Statistical Analysis

Data were analyzed using SPSS package version 25.0 (SPSS Inc, Chicago, IL, USA). The normal distribution of the sample was analyzed using the Shapiro–Wilk test, and a paired sample t-test was used to compare the means of pre-post measurements. Cohen’s d coefficient was used to determine sample effect size. The Chi Square correlation coefficient was used to evaluate the relationship between Multicomponent Physical Exercise Intervention and disability due to dyspnea and health status assessment at the 2-year follow-up, and *p* < 0.05 was considered statistically significant.

## 3. Results

The baseline characteristics of the 39 patients (mean age 63.85 ± 8.98 years; 29 male and 10 female) are listed in Table 1. 

Of the 39 patients recruited for the study, 25 (64%) had been discharged from ICU. We found no differences in the demographic variables at the baseline levels of the primary outcomes between patients from ICU and those from other services (duration of MEP, STR, COPD/CAT and 6MWT). Of all patients, 90% were somewhat–much worse than a year ago and felt moderate-to-intense dyspnea, and 79% presented a health status of somewhat–much worse than a year ago. The MEP treatment was 13.15 ± 2.62 sessions. Not all evaluations were collected for all patients during that time. Five patients did not complete the entire MEP, and no adverse effects were detected after the application of the treatments. The moderating effect of the demographic information of age and sex did not correlate with any of the outcomes of the present study.

### 3.1. Cardio-Respiratory Performance Assessment

At baseline assessment, patients presented a Steep Run Test (SRT) of 112.84 ± 49.52 (*p* = 0.097). In contrast, SRT after MEP increased to 188.39± 64, (*p* = 0.00 vs. pre-treatment; *n* = 28). Within-group effect sizes were greater at the post-treatment period (d > 0.8), as shown in Table 2.

### 3.2. Health Status Assessment

At baseline assessment, patients presented an SF-36 of 4.45 ± 0.6 over the five possible health states, 90% of them being somewhat–much worse than a year ago (*p* = 0.000). In contrast, SF-36 after MEP decreased to 3.82 ± 0.8 over the five possible health states (*p* = 0.005 vs. Pre-treatment; *n* = 22). Within-group effect sizes were negatively greater at the post-treatment period (d > −0.8), Table 2. 

### 3.3. Impact of COVID-19 on Daily Life Assessment

We found no significant differences for time in CAT/COPD, which had values of 17.29 ± 3.02 (*p* = 0.077) and 12.50 ± 8.66 (*p* = 0.075 vs. Pre-treatment; *n* = 14), respectively (Table 2). Within-group effect sizes were negatively moderate at the post-treatment period (d < −0.8), as shown in Table 2.

### 3.4. Disability Due to Dyspnea Assessment

At baseline assessment, patients presented an mMRC of 2.48 ± 0.98, with 79% of them experiencing moderate-to-intense dyspnea (*p* = 0. 002). In contrast, mMRC after MEP decreased to 1.62 ± 1.12, (*p* = 0.023 vs. Pre-treatment; *n* = 21). Within-group effect sizes were negatively greater at the post-treatment period (d > −0.8), Table 2. 

### 3.5. Aerobic Capacity and Endurance Assessment

Statistically significant differences over time were found in terms of the increase in oxygen saturation (95.23 ± 2.42 vs. 96.33 ± 1.83; *p* = 0.023 vs. Pre-treatment; *n* = 30), the decrease in resting heart rate (88.77 ± 15.29 vs. 83 ± 13.25; *p* = 0.033 vs. Pre-treatment; *n* = 31), the increase in the distance performed in the 6MWT (343 ± 107.8 vs. 444.55 ± 111.15; *p* = 0.000 vs. Pre-treatment; *n* = 31), and the decrease in the number of stops during the test (0.32 ± 0.54 vs. 0.03 ± 0.18; *p* = 0.005 vs. Pre-treatment; *n* = 31). Within-group effect sizes were negatively greater at the post-treatment period (d > −0.8), Table 3. 

### 3.6. Disability Due to Dyspnea and Health Status Assessment at the 2-Year Follow-Up 

**Figure 3:**
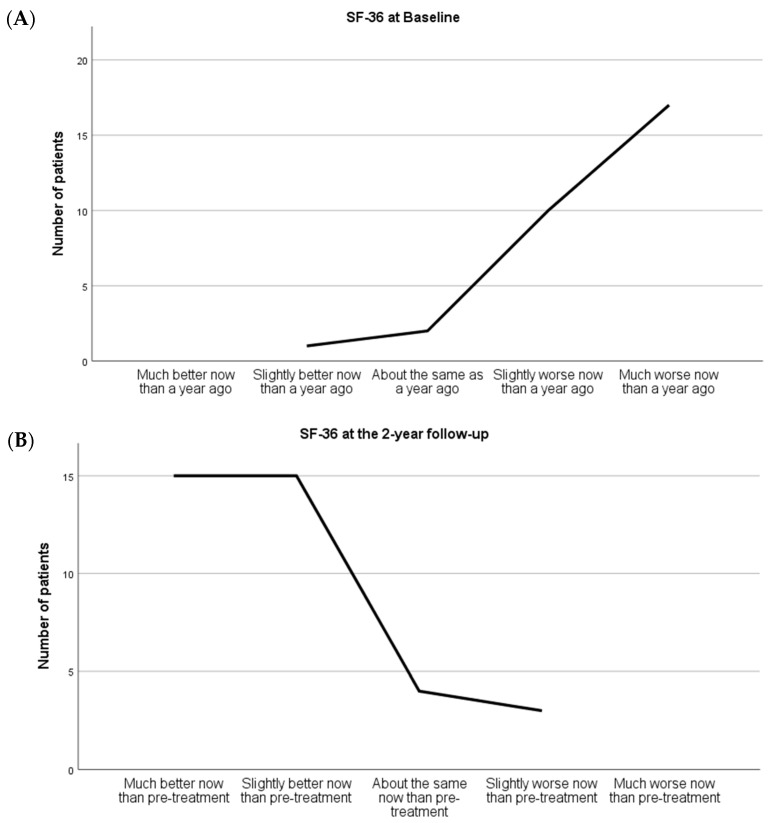
(**A**) Graphic of the results of the SF-36 Health Questionnaire at Baseline. (**B**) Graphic of the results of the SF-36 Health Questionnaire at the 2-year follow-up period 2-year follow-up period.

A total of 37 of the 39 patients completed the evaluation at the 2-year follow-up. Statistically significant differences over time were found in the increase in health status (*p* = 0.002), considering that 90% of patients presented at baseline a health status of “somewhat-much worse than a year ago”. At the 2-year follow-up, 81% of the patients were “much-slightly better now than pre-treatment” (*p* = 0.002 vs. Pre-treatment; *n* = 37) (Figure 3). 

**Figure 4:**
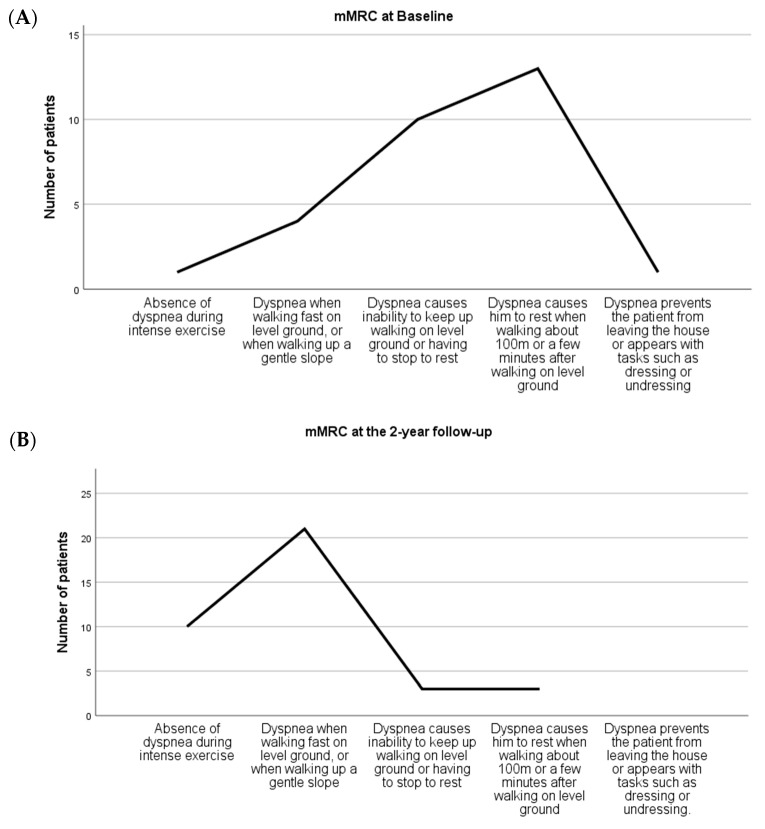
(**A**) Graphic of the results of the Medical Research Council dyspnea scale at Baseline. (**B**) Graphic of the results of the Medical Research Council dyspnea scale at the 2-year follow-up period.

Likewise, statistically significant differences over time were found in the reduction in disability due to dyspnea (*p* = 0.000), considering that 79% of patients presented a moderate-to-intense dyspnea status at baseline. At the 2-year follow-up, 84% of the patients experienced no dyspnea at all or only when walking fast on flat terrain, or when climbing a gentle slope (*p* = 0.000 vs. Pre-treatment; *n* = 37) (Figure 4). 

### 3.7. Post-COVID-19 Sequelae at the 2-Year Follow-Up 

**Figure 5:**
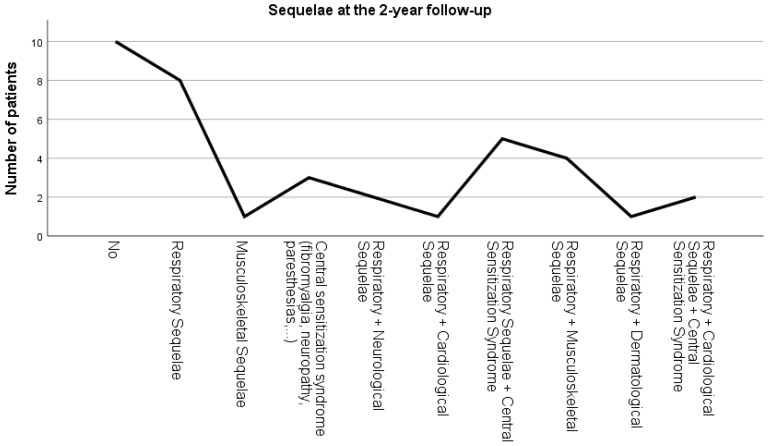
Signs and symptoms occurring at the 2-year follow-up period.

At 2-year follow-up, 27% of the 37 patients had no sequelae after COVID-19, 22% presented respiratory sequelae, 13% presented respiratory sequelae and central sensitization syndrome, 11% presented respiratory and musculoskeletal sequelae, 8% signs and symptoms of central sensitization, 5% presented respiratory and cardiological sequelae and central sensitization syndrome, 5% presented respiratory and neurological sequelae, 3% presented respiratory and cardiologic sequelae, as well as musculoskeletal (3%), and respiratory and dermatological (3%) (Figure 5) sequalae. None of the patients reported any limitations in daily life, and 32 (86%) continued to exercise at discharge.

## 4. Discussion

The result of the present study shows the positive effects of the MEP at the pre-post assessment and the 2-year follow-up on improving the immediate sequelae of post-COVID-19 patients. Of the 39 patients recruited for the study, 25 (64%) had been discharged from ICU, and no adverse effects were detected after the application of the treatments. This indicates that MEP is a safe intervention for post-COVID patients, also considering its low cost. The results of this study show a significant improvement in disability due to dyspnea and in aerobic capacity and endurance after intervention; and an increase in health status and reduction in disability due to dyspnea at the 2-year follow-up. To our knowledge, this is the first study with a long-term follow-up in the field addressing immediate post COVID-19 sequelae with a MEP. 

### 4.1. Functional Capacity

In relation to cardio-respiratory performance, patients achieved an increase in SRT following 7 weeks of the multicomponent program. However, there has been no study analyzing the effects with this assessment tool in this group of patients; however, other authors have extensively studied this tool when evaluating aerobic capacity in patients with multisystemic involvement such as oncology ones [30]. Weemaes et al. [30] concluded that SRT is correlated with the cardiopulmonary exercise test—VO_2_ peak; therefore, it seems this tool is a valid one with which to estimate aerobic capacity and our study could provide a precedent for use in patients with cardiopulmonary impairment, as we provided an evidence-based study for clinical practice. 

In connection with exercise capacity, Udina et al. [17] conducted a study with a similar sample size to the one in this intervention, using the 6MWT to assess walked distance. This measure improved from 158.7 ± 154.1 to 346.3 ± 111.5 m, a significantly different within-group result was also found in our study with an improvement from 343 ± 107.8 to 444.55 ± 111.15 m. Likewise, Curci et al. [20] also included a similar sample size but patient’s condition limited the assessment through this test to a small number of patients obtaining poor results. This research team used the 6MWT to analyze those parameters that are relevant in the clinical setting and that indicate the patient’s baseline condition and response to exercise, reflecting significant differences with an increase in the oxygen saturation and a decrease in resting heart rate. The study conducted by Udina [17] and Curci [20] did not mention the analysis of these physiological variables. Ferioli et al. [27] concluded that 6MWT is a useful test in post-COVID follow-up, correlating with the severity of acute phase and impairments in the chronic one, offering the possibility to assess improvements in exercise capacity. Because of this conclusion and the limited but significant results obtained in the present study, this tool should be taken into account in further studies involving post-COVID patients due to its low cost and the great variability of the relevant data it provides. 

### 4.2. Health Status 

The results of this study do not reflect significant differences for time in CAT/COPD. Daynes et al. [24], however, concluded that CAT is a useful tool to assess symptoms of COVID-19 recovery in a study of 131 patients (one-third of which had a pre-existing respiratory condition); however, they took into consideration that its application in post-COVID-19 patients has to be accompanied by an assessment of psychosocial factors in relation to symptom perception and expectations of benefits from the treatment to be received. Future research with post-COVID-19 patients should be oriented towards a global assessment of health status, while taking into consideration an inactive perspective where social and psychological factors could be of great relevance, which Daher et al. took into account [31]. 

SF-36 results were statistically significant in this stud. These outcomes are supported by a structured review wherein Poudel et al. [23] concluded that the impact of COVID-19 on the health-related quality of life of acute COVID-19 patients compared to long COVID-19 patients was higher; and that impacts were higher among severe patients admitted to ICU compared to those who were admitted to general wards. 

### 4.3. Disability Due to Dyspnea 

The results of this study determined that disability due to dyspnea after MEP decreases from 2.48 pre-intervention to 1.62 post-intervention. Curci et al. [20] provided an accurate description of sub-acute patients admitted to the rehabilitation unit in a sample similar to ours (32 patients) obtaining scores between 4 and 5 on this scale, which shows a worse baseline situation of the patients. 

The basal situation of patients could have been conditioned by the medication used during different waves to limit the acute conditions that led them to the ICU. These drugs included the following: Cyclophosphamide, Interferon beta, Kaletra, Dolquine, Tocilizumab and corticosteroids in the first wave and only corticosteroids from the second one [32]. 

### 4.4. Limitations 

An important limitation of the study is the small sample size. Also important is the absence of a control group or placebo group to compare with the evolution of the process or with other interventions. 

Although there is an inherent bias in the quality of the information presented in this type of study, this has been minimized by using relevant and reliable resources. 

## 5. Conclusions

Individualized and monitored MEP in survivors of COVID-19 showed positive effects in cardio-respiratory performance, health status, disability due to dyspnea, and aerobic capacity and endurance at pre-post treatment. 

Furthermore, an increase in health status and an overall reduction in disability due to dyspnea was achieved in all post COVID-19 patients at 2-year follow up, improving the immediate sequelae. 

Furthermore, none of the patients had any adverse effects either pre-post treatment or at the 2-year follow-up. 

This highlights the importance of the professional background of the rehabilitation teams in adapting to an unknown clinical environment. 

## Figures and Tables

**Table 1:** Baseline patient characteristics. Values are means ± standard deviation (95% confidence interval). *p* < 0.05 is statistically significant.

Characteristic	MEP (Pre)	SD	*p*
N	39		
Age (years)	63.85	±8.98	0.126
Sex M/F (Male %)	29/10 (67.4%)		
Previous ICU stay (Yes, %)	25 (58%)		
SF-36 (% of somewhat-much worse)	27 (90%)		
mMRC (% moderate-to-intense dyspnea)	25 (79%)		
Duration of MEP (sessions)	13.15	±2.62	0.248
Weight (Kg)	78.52	±16.37	0.564
Height (cm)	167.61	±9.22	0.567
BMI	27.7	±4.82	0.379
SRT (Watts)	112.84	±49.52	0.097
COPD Assessment Test (CAT)	15.30	±6.52	0.077
6MWT			
Oxygen Saturation (%)	95.14	±2.95	0.006 #
Heart Rate (BPM)	89	±15.15	0.846
Blood Pressure (SBP/DBP; mmHg)	130.85/76	±14.87/±11.4	0.7/0.8
Distance (m)	343.25	±102.61	0.315
Number of stops during the test	0.31	±0.52	0.00 #
Borg RPR	2.67	±2.15	0.81

# Significantly different within-group, *p* < 0.05 (95% confidence interval); Abbreviations: ICU, Intensive Care Unit; SF-36, Health Questionnaire Short Form-36 (% excluding missing values); mMRC, Modified Medical Research Council (% excluding missing values); MEP, Multicomponent Exercise Program; BMI, Body Mass Index; STR, Steep Run Test; CAT/COPD, Chronic Obstructive Pulmonary Disease Assessment Test (scores over 40; higher scores result in poorer outcomes); 6MWT, 6 min Walk Test; BPM, Beats Per Minute; SBP/DBP, Systolic Blood Pressure and Diastolic Blood Pressure; mmHg, millimeters of Mercury; Borg RPR, Borg Rating of Perceived Exertion Scale; SD, Standard Deviation.

**Table 2:** Adjusted means (SD) for outcome at all study visits and mean (SD) difference within group and effect size.

**STR (Watts)**			
**Means (SD)**		**Difference within Group Post Minus Pre (*n* = 39)**	**Effect Size Cohen’s d**
**MEP (Pre)**	**MEP (Post)**		
119.64 ± 52.42	188.39 ± 64	−68.75 (−84.05; −53.45) *	1.17
**SF-36**			
**Means (SD)**		**Difference within Group Post Minus Pre (*n* = 39)**	**Effect Size Cohen’s d**
**MEP (Pre)**	**MEP (Post)**		
4.45 ± 0.6	3.82 ± 0.8	0.64 (0.21; 1.06) #	−0.89
**COPD Assessment Test (CAT)**			
**Means (SD)**		**Difference within Group Post Minus Pre (*n* = 39)**	**Effect Size Cohen’s d**
**MEP (Pre)**	**MEP (Post)**		
17.29 ± 3.02	12.50 ± 8.66	4.79 (−0.55; 10.12)	−0.74
**Modified Medical Research Council (mMRC)**			
**Means (SD)**		**Difference within Group Post Minus Pre (*n* = 39)**	**Effect size Cohen’s d**
**MEP (Pre)**	**MEP (Post)**		
2.48 ± 0.98	1.62 ± 1.12	0.86 (0.13; 1.58) #	−0.82

# Significantly different within-group, *p* < 0.05 (95% confidence interval); * Significantly different within-group, *p* < 0.001 (95% confidence interval). Abbreviations: MEP, Multicomponent Exercise Program; STR, Steep Run Test (incremental field test in cycloergometer); SF-36, Health Questionnaire Short Form-36; CAT/COPD, Chronic Obstructive Pulmonary Disease Assessment Test (scores over 40; higher scores result in poorer outcomes); SD, Standard Deviation.

**Table 3:** Adjusted means (SD) for outcome at all study visits and mean (SD) difference within group and effect size.

6MWT				
	Means (SD)		Difference within Group Post Minus Pre (*n* = 39)	Effect Size Cohen’s d
	MEP (Pre)	MEP (Post)		
Oxygen Saturation (%)	95.23 ± 2.42	96.33 ± 1.83	−1.11 (−2.04; −0.16) #	0.51
Heart Rate (BPM)	88.77 ± 15.29	83 ± 13.25	5.77 (0.48; 11.07) #	−0.4
Blood Pressure	130.4 ± 15.5	128.5 ± 17	1.86 (−3.34; 7.05)	−0.18
(SBP/DBP; mmHg)	77.1 ± 11.6	77.5 ± 10.1	−0.4 (−4.2; 3.41)	0.04
Distance (m)	343 ± 107.8	444.55 ± 111.15	−101.6 (−135.12; −68.04) *	0.93
Number of stops during the test	0.32 ± 0.54	0.03 ± 0.18	0.29 (0.1; 0.48) #	−0.72
Borg RPR	2.61 ± 2.03	2.19 ± 2.14	0.42 (−0.47; 1.31)	−0.2

# Significantly different within-group, *p* < 0.05 (95% confidence interval); * Significantly different within-group, *p* < 0.001 (95% confidence interval). Abbreviations: MEP, Multicomponent Exercise Program; 6MWT, 6-min Walk Test; BPM, Beats Per Minute; mmHg, millimeters of Mercury; Borg RPR, Borg Rating of Perceived Exertion Scale; SD, Standard Deviation.

## Data Availability

The data presented in this study are available on request from the corresponding authors. The data are not publicly available due to ethical restrictions.

## References

[1] Barker-Davies R.M., O’Sullivan O., Senaratne K.P.P., Baker P., Cranley M., Dharm-Datta S., Ellis H., Goodall D., Gough M., Lewis S. (2020). The Stanford Hall consensus statement for post-COVID-19 rehabilitation. Br. J. Sports Med..

[2] Sánchez Romero E.A., Rolando L.M., Villafañe J.H. (2022). Impact of Lockdown on Patients with Fibromyalgia. Electron. J. Gen. Med..

[3] Long B., Carius B.M., Chavez S., Liang S.Y., Brady W.J., Koyfman A., Gottlieb M. (2022). Clinical update on COVID-19 for the emergency clinician: Presentation and evaluation. Am. J. Emerg. Med..

[4] Reviriego G.B., Pascual B.P., Ruiz A.R., Sánchez Romero E.A., Corbelini C., Villafañe J.H. (2020). Spanish Experience of Pulmonary Rehabilitation Efficacy for Patients Affected by the Novel SARS-CoV-2 (COVID-19): A Case Report. Top. Geriatr. Rehabil..

[5] Sánchez Romero E.A., Pérez J.L.A., Suárez I.V., Corbellini C., Villafañe J.H. (2022). Spanish experience on the efficacy of airways clearance techniques in SARS-CoV-2 (COVID-19) at intensive care unit: An editorial and case report. SAGE Open Med. Case Rep..

[6] Fiore E., Corbellini C., Acucella L., Gargano S., Sánchez Romero E.A., Cotella D., Villafañe J.H. (2022). Musculoskeletal pain related to COVID-19 survivors after hospitalization: A short review (Dolor musculoesquelético en supervivientes del COVID-19 tras la hospitalización: Una breve revision). Retos.

[7] Corbellini C., Villafane J., Gugliotta E., Tavella S., Zampese S., Pessina P., Monti R., Carnuccio C., Sánchez Romero E.A., Meroni R. (2021). Late Breaking Abstract–Pulmonary rehabilitation in post–COVID subjects with moderate lung restriction, a case series. Eur. Respir. J..

[8] Talman S., Boonman-de Winter LJ M., De Mol M., Hoefman E., Van Etten R.W., De Backer I.C. (2021). Pulmonary function and health-related quality of life after COVID-19 pneumonia. Respir. Med..

[9] Peeling R.W., Heymann D.L., Teo Y.Y., Garcia P.J. (2022). Diagnostics for COVID-19: Moving from pandemic response to control. Lancet.

[10] Chilamakuri R., Agarwal S. (2021). COVID-19: Characteristics and Therapeutics. Cells.

[11] Goodwin V.A., Allan L., Bethel A., Cowley A., Cross J.L., Day J., Drummond A., Hall A.J., Howard M., Morley N. (2021). Rehabilitation to enable recovery from COVID-19: A rapid systematic review. Physiotherapy.

[12] de Sire A., Andrenelli E., Negrini F., Patrini M., Lazzarini S.G., Ceravolo M.G., The International Multiprofessional Steering Committee of Cochrane Rehabilitation REH-COVER Action (2020). Rehabilitation and COVID-19: The Cochrane Rehabilitation 2020 rapid living systematic review. Eur. J. Phys. Rehabil. Med..

[13] Corbellini C., Rossino E., Massaccesi R., Battaglino A., Pedersini P., Sánchez Romero E.A., Villafañe J.H. (2022). Improvements in Perimeter Thoracic Mobility on Patients with COPD after Pulmonary Rehabilitation: A Case Series. Electron. J. Gen. Med..

[14] Neder J.A., Marillier M., Bernard A.-C., James M.D., Milne K.M., O’Donnell D.E. (2019). The Integrative Physiology of Exercise Training in Patients with COPD. COPD: J. Chronic Obstr. Pulm. Dis..

[15] Schulté B., Nieborak L., Leclercq F., Villafañe J.H., Sánchez Romero E.A., Corbellini C. (2022). The Comparison of High-Intensity Interval Training Versus Moderate-Intensity Continuous Training after Coronary Artery Bypass Graft: A Systematic Review of Recent Studies. J. Cardiovasc. Dev. Dis..

[16] Jimeno-Almazán A., Pallarés J., Buendía-Romero A., Martínez-Cava A., Franco-López F., Martínez B.S.-A., Bernal-Morel E., Courel-Ibáñez J. (2021). Post-COVID-19 Syndrome and the Potential Benefits of Exercise. Int. J. Environ. Res. Public Health.

[17] Udina C., Ars J., Morandi A., Vilaró J., Cáceres C., Inzitari M. (2021). Rehabilitation in adult post-COVID-19 patients in post-acute care with Therapeutic Exercise. J. Frailty Aging.

[18] Cuenca-Zaldivar J.N., Acevedo M., Fernández-Carnero J., Sánchez-Romero E.A., Villafañe J.H., Carballar C.B. (2022). Effects of a Multicomponent Exercise Program on Improving Frailty in Post-COVID-19 Older Adults after Intensive Care Units: A Single-Group Retrospective Cohort Study. Biology.

[19] Global Burden of Disease 2020 Health Financing Collaborator Network (2021). Tracking development assistance for health and for COVID-19: A review of development assistance, government, out-of-pocket, and other private spending on health for 204 countries and territories, 1990–2050. Lancet.

[20] Curci C., Pisano F., Bonacci E., Camozzi D.M., Ceravolo C., Bergonzi R., De Franceschi S., Moro P., Guarnieri R., Ferrillo M. (2020). Early rehabilitation in post-acute COVID-19 patients: Data from an Italian COVID-19 Rehabilitation Unit and proposal of a treatment protocol. Eur. J. Phys. Rehabilitation Med..

[21] Sánchez Romero E.A., Carnero J.F., Pérez J.L.A., Rolando L.M., Villafañe J.H. (2022). Addressing post-COVID-19 musculoskeletal symptoms through telemedicine: A study protocol. F1000Research.

[22] Yasui H., Inui N., Karayama M., Mori K., Hozumi H., Suzuki Y., Furuhashi K., Enomoto N., Fujisawa T., Nakamura Y. (2019). Correlation of the modified Medical Research Council dyspnea scale with airway structure assessed by three-dimensional CT in patients with chronic obstructive pulmonary disease. Respir. Med..

[23] Poudel A.N., Zhu S., Cooper N., Roderick P., Alwan N., Tarrant C., Ziauddeen N., Yao G.L. (2021). Impact of COVID-19 on health-related quality of life of patients: A structured review. PLoS ONE.

[24] Daynes E., Gerlis C., Briggs-Price S., Jones P., Singh S.J. (2021). COPD assessment test for the evaluation of COVID-19 symptoms. Thorax.

[25] Zhou Z., Zhou A., Zhao Y., Chen P. (2017). Evaluating the Clinical COPD Questionnaire: A systematic review. Respirology.

[26] Kendrick K.R., Baxi S.C., Smith R.M. (2000). Usefulness of the modified 0–10 Borg scale in assessing the degree of dyspnea in patients with COPD and asthma. J. Emerg. Nurs..

[27] Ferioli M., Prediletto I., Bensai S., Betti S., Daniele F., Scioscio V.D., Modolon C., Rimondi M.R., Nava S., Fasano L. (2021). The role of 6MWT in COVID-19 follow up. Paper presented at: ERS 2021. The European Respiratory Society International Congress. Eur. Respir. J..

[28] Simonelli C., Paneroni M., Vitacca M., Ambrosino N. (2021). Measures of physical performance in COVID-19 patients: A mapping review. Pulmonology.

[29] Werkman M.S., Bongers B.C., Blatter T., Takken T., Wittink H. (2020). Extended steep ramp test normative values for 19–24-year-old healthy active young adults. Eur. J. Appl. Physiol..

[30] Weemaes A.T., Beelen M., Bongers B.C., Weijenberg M.P., Lenssen A.F. (2021). Criterion Validity and Responsiveness of the Steep Ramp Test to Evaluate Aerobic Capacity in Survivors of Cancer Participating in a Supervised Exercise Rehabilitation Program. Arch. Phys. Med. Rehabil..

[31] Daher A., Balfanz P., Cornelissen C., Müller A., Bergs I., Marx N., Müller-Wieland D., Hartmann B., Dreher M., Müller T. (2020). Follow up of patients with severe coronavirus disease 2019 (COVID-19): Pulmonary and extrapulmonary disease sequelae. Respir. Med..

[32] Scavone C., Brusco S., Bertini M., Sportiello L., Rafaniello C., Zoccoli A., Berrino L., Racagni G., Rossi F., Capuano A. (2020). Current pharmacological treatments for COVID-19: What’s next?. Br. J. Pharmacol..

